# Tryptophan Metabolism in Patients With Chronic Kidney Disease Secondary to Type 2 Diabetes: Relationship to Inflammatory Markers

**DOI:** 10.1177/1178646917694600

**Published:** 2017-03-10

**Authors:** Subrata Debnath, Chakradhar Velagapudi, Laney Redus, Farook Thameem, Balakuntalam Kasinath, Claudia E Hura, Carlos Lorenzo, Hanna E Abboud, Jason C O’Connor

**Affiliations:** 1 Division of Nephrology, Department of Medicine, University of Texas Health Science Center at San Antonio, San Antonio, TX, USA; 2 Department of Pharmacology, University of Texas Health Science Center at San Antonio, San Antonio, TX, USA; 3 Division of Rheumatology and Clinical Immunology, Department of Medicine, University of Texas Health Science Center at San Antonio, San Antonio, TX, USA; 4 South Texas Veterans Health Care System, San Antonio, TX, USA

**Keywords:** Chronic kidney disease, indoleamine 2,3-dioxygenase 1, inflammatory cytokines, kynurenine, tryptophan, type 2 diabetes

## Abstract

**Objective:**

Type 2 diabetes (T2D) is the primary case of chronic kidney disease (CKD). Inflammation is associated with metabolic dysregulation in patients with T2D and CKD. Tryptophan (TRP) metabolism may have relevance to the CKD outcomes and associated symptoms. We investigated the relationships of TRP metabolism with inflammatory markers in patients with T2D and CKD.

**Methods:**

Data were collected from a well-characterized cohort of type 2 diabetic individuals with all stages of CKD, including patients on hemodialysis. Key TRP metabolites (kynurenine [KYN], kynurenic acid [KYNA], and quinolinic acid [QA]), proinflammatory cytokines (tumor necrosis factor-α [TNF-α] and interleukin-6 [IL-6]), and C-reactive protein were measured in plasma. The KYN/TRP ratio was utilized as a surrogate marker for indoleamine 2,3-dioxygenase 1 (IDO1) enzyme activity.

**Results:**

There was a significant inverse association between circulating TRP level and stages of CKD (*P* < 0.0001). Downstream bioactive TRP metabolites KYN, KYNA, and QA were positively and robustly correlated with the severity of kidney disease (*P* < 0.0001). In multiple linear regression, neither TNF-α nor IL-6 was independently related to KYN/TRP ratio after adjusting for estimated glomerular filtration rate (eGFR). Only TNF-α was independently related to KYN after taking into account the effect of eGFR.

**Conclusions:**

Chronic kidney disease secondary to T2D may be associated with accumulation of toxic TRP metabolites due to both inflammation and impaired kidney function. Future longitudinal studies to determine whether the accumulation of KYN directly contributes to CKD progression and associated symptoms in patients with T2D are warranted.

## Introduction

Type 2 diabetes (T2D) is the single most important cause of chronic kidney disease (CKD), which often progresses to end-stage kidney disease (ESKD).^1^ In patients with T2D, several factors play mechanistic roles in the initiation of CKD and progression to ESKD. Patients with advanced CKD endure myriad symptoms and dismal health outcomes. Inflammation has emerged as a novel risk marker in relation to the chronic worsening of kidney function and clinical complications in CKD.^2^ Metabolic aberrations, e.g. dysregulation in amino acid metabolism, are characteristics of CKD.^3,4^ Chronic inflammatory response is also associated with significant metabolic activity with consequent nutrient depletion in CKD.^4^

**Figure 1.**
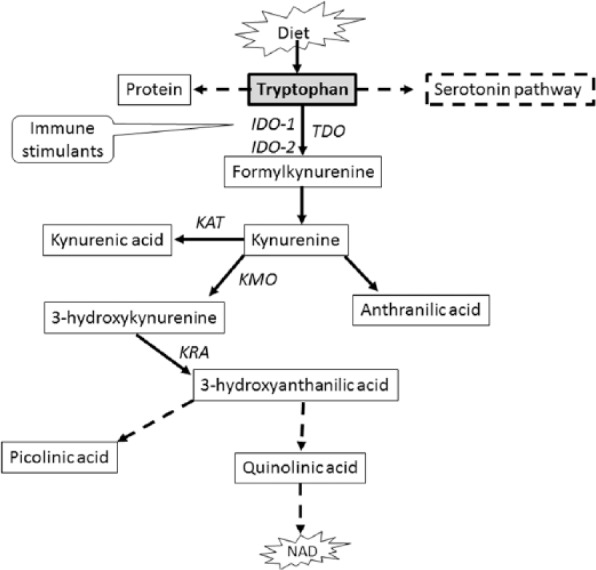
Simplified pathway of tryptophan metabolism in mammals. Dashed arrow indicates multiple processes. Solid arrow indicates single process. IDO-1, indoleamine 2,3-dioxygenase 1; IDO-2, indoleamine 2,3-dioxygenase 2; KAT, kynurenine aminotransferase; KMO, kynurenine 3-monooxygenase; KRA, kynureninase; NAD, nicotinamide adenine dinucleotide; TDO, tryptophan 2,3-dioxygenase.

Essential amino acid l-tryptophan (TRP) contributes to the synthesis of nicotinamide adenine dinucleotide, a coenzyme important for energy metabolism in the mammalian tissues (Figure 1).^5,6^ The majority (~95%) of free TRP undergoes oxidative metabolism along the kynurenine (KYN) pathway yielding KYN involving 2 key enzymes: tryptophan 2,3-dioxygenase (TDO, highly expressed in the liver) and indoleamine 2,3-dioxygenase 1 (IDO1), which is expressed extrahepatically. Indoleamine 2,3-dioxygenase 1, potently induced by proinflammatory cytokines,^7-9^ acts locally to modulate TRP levels in response to inflammation. Kynurenine metabolism eventually generates kynurenic acid (KYNA) and other bioactive metabolites 3-hydroxykynurenine, quinolinic acid (QA), etc. collectively known as kynurenines. The regulation of TRP metabolism is sensitive to environmental stimuli (e.g. inflammation), and kynurenines are normally eliminated via urinary excretion. Because kynurenines play an important role in regulation of adaptive immunity and are implicated in comorbid atherosclerosis and neuropsychiatric symptoms, patients with CKD are at particularly high risk of KYN-associated pathophysiologies.^10^ For example, altered TRP metabolism may precipitate fatigue in patients with CKD.^11^ Likewise, TRP metabolites (e.g. KYN and QA) may promote atherosclerosis in ESKD by activating oxidative stress and leukocyte activation in endothelial and vascular smooth muscle cells.^12^

The kidneys play an integral role in the metabolism of TRP.^13^ The rate-limiting enzyme IDO1 is overexpressed in kidney tissues.^14^ Interestingly, aberrations in TRP metabolism and specific enzyme activities in the KYN pathway could contribute to the pathogenesis of T2D.^15^ It should be emphasized that the molecular mechanisms of inflammatory pathways are also different in nondiabetic vs diabetic CKD,^16^ the latter group having an accelerated loss of kidney function than the former.^17^ Therefore, TRP metabolism may have potentially important clinical implications to CKD secondary to T2D. The association between inflammatory cytokines and TRP metabolism across the stages of CKD in patients with T2D is unknown. We aimed to study the associations between TRP metabolism and stages of CKD in presence of inflammatory markers in patients with T2D.

## Materials and Methods

### Participants

We analyzed data collected from 60 type 2 diabetic patients who participated in the Family Investigation of Nephropathy in Diabetes (FIND) study. The FIND study design, population, and phenotypic data have been published previously.^18^ Briefly, participants had clinical diagnosis of T2D^19^ and CKD, including ESKD (Supplemental Table).^20^ All participants were receiving standard medical treatment for diabetes, CKD, and ESKD and associated comorbidities as per guidelines.^20,21^ Individuals with chronic inflammatory conditions such as chronic hepatitis and rheumatoid arthritis were excluded. All participants provided written consent to the protocol approved by the Institutional Review Board at the University of Texas Health Science Center at San Antonio. Estimation of glomerular filtration rate (eGFR) was calculated using the Modification of Diet in Kidney Disease equation.^22^ The KYN/TRP ratio was calculated to estimate IDO1 enzyme activity.^23^

### Laboratory measurements and inflammatory markers

Fasting blood was collected from each participant and stored at −80°C until analyzed. Creatinine and albumin were measured in stored serum using standard methods by the centralized laboratory. Albumin and creatinine in urine were quantified in all subjects except subjects with CKD stage 5 on ESKD. Plasma interleukin-6 (IL-6) and tumor necrosis factor-α (TNF-α) were measured by high-sensitive enzyme-linked immunosorbent assay (Quantikine HS Human Immunoassay; R&D Systems, Minneapolis, MN) according to the manufacturer’s instructions. The lower detection limits for IL-6 and TNF-α were 0.19 pg/mL and 0.11 pg/mL with intra-assay coefficients of variation (CV) 3.1% to 8.7% and 5.5% to 9.8% and inter-assay CV 7.4% to 10.4% and 5.5% to 11.2%, respectively. C-reactive protein (CRP) levels were measured by rate immunoturbidimetry (Beckman Coulter Inc., Brea, CA, USA) using a high-sensitivity CRP assay (lower detection limit of 0.2 mg/L).

### TRP metabolites

Tryptophan and selective metabolites in the KYN pathway were measured in plasma by liquid chromatography/mass spectrometry (LC-MS) as reported previously.^24^ Briefly, 50µL plasma was diluted with 5 times of 0.2% acetic acid. Stable isotope–labeled standards, 2-picolinic-d_4_ acid, 2,3-pyridinedicarboxylic acid-d_3_, L-TRP-13C_11_,15N_2_, and KYN, were added at the time of extraction as internal standards for absolute quantification. The diluted samples were vortexed and transferred to 0.5-mL Millipore Amicon Ultra filter (3 kDa). The filter tubes were centrifuged at 13 500*g* for 60 minutes at 4°C and the extracts were transferred to glass vials for LC-MS analyses. High-performance liquid chromatography/electrospray ionization mass spectrometry (HPLC-ESI-MS) analyses were conducted on a Thermo Fisher Q Exactive mass spectrometer with online separation by a Thermo Fisher/Dionex Ultimate 3000 HPLC. High-performance liquid chromatography conditions were as follows: column, YMC-Pack ODS-AQ, 3 µm, 2 × 100 mm (YMC; Allentown, PA); mobile phase A, 0.5% formic acid in water; mobile phase B, 1% formic acid in acetonitrile; flow rate, 200 µL/min; gradient, 1% B to 30% B for 5 minutes and held at 70% B for 5 minutes to clean the column. The MS analyses were conducted using full MS scan (70 000 resolution) with positive ion detection. Standard curves were generated for all targeted KYN compounds using appropriate stable isotope–labeled internal standards and native compounds. Quantitative results were obtained by reference of the experimental peak area ratios to the standard curves.

### Statistical analysis

Statistical analyses were performed using SAS statistical software (version 9.2, SAS Institute Inc, Cary, NC) and R Project statistical software packages (version 2.9.2; the R Foundation for Statistical Computing, Vienna, Austria). Variables were expressed as mean ± standard deviation or 95% confidence interval as appropriate and percentages as appropriate. Continuous variables were tested for skewness and kurtosis, and some of them were log transformed as appropriate to approximate normality. Spearman rho correlations were used to assess simple bivariate associations. We used one-way analysis of covariance (ANCOVA) to examine associations between variables of interest to account for the effects of potential confounding variables. Multiple linear regression models were used to analyze KYN/TRP ratio (IDO1 activity) association with inflammatory markers. We assessed the relationship of TRP metabolites to eGFR modeled by a smooth function with the SemiPar 1.0 R package. We considered a two-sided *P* value less than 0.05 to be statistically significant. The Bonferroni correction was used for multiple group comparisons as applicable.

## Results

Demographic and laboratory characteristics of the participants are presented in Table 1. The mean body mass index (BMI, calculated using weight in kilograms over height in meters squared) of the study participants was 30.83 ± 7.18, categorizing them as obese. The mean BMI of the patients in stage 5 CKD, however, was significantly (*P* < 0.05) lower than that of the mean BMI observed in CKD stages 1 to 4. The mean eGFR of the study participants was 51.26 ± 43 mL/min/1.73 m^2^.

**Table 1.** Characteristics of the study participants (n = 60).

Variable	
Age, y	57.17 ± 11.84
Sex (female)	33 (55%)
Body mass index, kg/m^2^	30.83 ± 7.18
Serum albumin, g/dL	3.61 ± 0.51
eGFR, mL/min/1.73 m^2a^	51.26 ± 43
CRP, mg/L	0.75 ± 0.86
TNF-α, pg/mL	5.71 ± 3.98
IL-6, pg/mL	3.20 ± 2.90
Tryptophan, µM	49.17 ± 20.24
Kynurenine, µM	3.80 ± 2.12
Kynurenic acid, µM	12.36 ± 4.25
3-Hydroxykynurenine, µM	0.12 ± 0.14
Quinolinic acid, µM	3.66 ± 4.10

Abbreviations: CRP, C-reactive protein; eGFR, estimated glomerular filtration rate; IL-6, interleukin-6; TNF-α, tumor necrosis factor-α.

Data are mean ± standard deviation or number (%).

a Excludes subjects (n = 21) with stage 5 CKD on hemodialysis.

Table 2 shows biochemical parameters, proinflammatory cytokines, and TRP metabolites by CKD stages. Serum albumin levels were within the recommended range for all CKD stages except stage 4. Among inflammatory markers, only TNF-α concentrations were markedly increased in stage 5 CKD compared with stage 1 CKD (*P* < 0.05). However, the *P* value did not reach statistical significance (*P* = 0.056) after Bonferroni correction. Likewise, plasma TRP levels were significantly lower (*P* < 0.0001) with concomitantly and significantly higher levels of its metabolites (e.g., KYN, KYNA, and QA—*P* < 0.001 for all) in advanced CKD than that of stage 1 CKD. The KYN/TRP ratio was robustly elevated (*P* < 0.0001) in CKD stages 4 and 5 compared with stage 1. After the Bonferroni correction, significance retained for TRP, KYN, KYNA, QA, and KYN/TRP ratio (*P* < 0.001 for all) in CKD stage 5 compared with stage 1.

**Table 2.** Age- and sex-adjusted body mass index and biochemical parameters by stages of chronic kidney disease.

Variable	Stage 1: eGFR ⩾90 (n = 12)	Stage 2: eGFR 60-89 (n = 15)	Stage 3: eGFR 30-59 (n = 8)	Stage 4: eGFR 15-29 (n = 4)	Stage 5 on ESKD: eGFR <15 (n = 21)
BMI, kg/m^2^	32.14 (28.57–36.15)	30.57 (27.72–33.72)	29.08 (25.85–32.71)	35.16 (29.48–41.95)	27.66^a^ (25.57–29.92)
Albumin, g/dL	3.78 (3.43–4.17)	3.74 (3.46–4.05)	3.63 (3.23–4.09)	3.16^a^ (2.75–3.62)	3.42 (3.23–3.63)
CRP, mg/L	0.43 (0.21–0.87)	0.30 (0.20–0.58)	0.26 (0.10–0.87)	0.29 (0.09–1.00)	0.68 (0.43–1.06)
TNF-α, pg/mL	3.16 (1.79–5.58)	2.41 (1.54–3.78)	3.74 (2.04–6.87)	6.89 (2.91–16.3)	7.69^a^ (5.30–11.1)
IL-6, pg/mL	2.39 (1.55–3.67)	1.65 (1.18–2.30)	1.03^a^ (0.66–1.62)	2.48 (1.30–4.74)	4.01 (3.05–5.28)
TRP, µM	69.4 (59.3–81.2)	58.6 (51.0–67.2)	50.4^a^ (42.3–60.1)	38.9^c^ (30.1–50.1)	28.2^d^ (25.1–31.7)
KYN, µM	2.08 (1.71–2.52)	2.53 (2.17–2.96)	3.32^b^ (2.68–4.12)	3.16^a^ (2.31–4.32)	5.58^d^ (4.87–6.41)
KYNA, µM	0.33 (0.20–0.53)	0.31 (0.21–0.46)	0.43 (0.25–0.73)	0.42 (0.20–0.88)	4.30^d^ (3.09–6.01)
3-HKYN, µM	1.05 (0.99–1.11)	1.06 (1.02–1.10)	1.06 (1.00–1.13)	1.12 (1.03–1.21)	1.22^d^ (1.17–1.27)
QA, µM	1.57 (1.24–1.98)	1.63 (1.37–1.95)	1.86 (1.47–2.35)	2.32 (1.66–3.23)	6.30^d^ (5.38–7.37)
KYN/TRP ratio	0.03 (0.03–0.04)	0.05^b^ (0.04–0.05)	0.07^d^ (0.05–0.08)	0.08^d^ (0.06–0.12)	0.20^d^ (0.18–0.22)

Abbreviations: 3-HKYN, 3-hydroxykynurenine; BMI, body mass index; CRP, C-reactive protein; eGFR, estimated glomerular filtration rate; ESKD, end-stage kidney disease; IL-6, interleukin-6; KYN, kynurenine; KYNA, kynurenic acid; QA, quinolinic acid; TNF-α, tumor necrosis factor-α; TRP, tryptophan.

Data are geometric means and 95% confidence interval. Variables (except BMI and albumin) are log transformed.

a *P* < 0.05; ^b^*P* < 0.01; ^c^*P* < 0.001; ^d^*P* < 0.0001.

**Figure 2.**
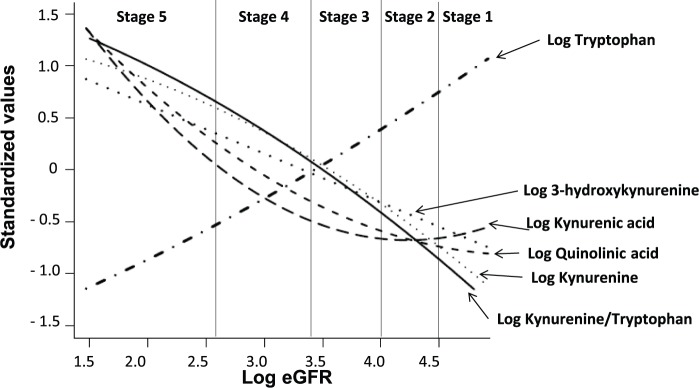
Relationship between kidney function and tryptophan metabolites.^a^ ^a^Penalized smoothing splines were used to assess the relationship between eGFR to tryptophan metabolites. eGFR indicates estimated glomerular filtration rate.

**Figure 3.**
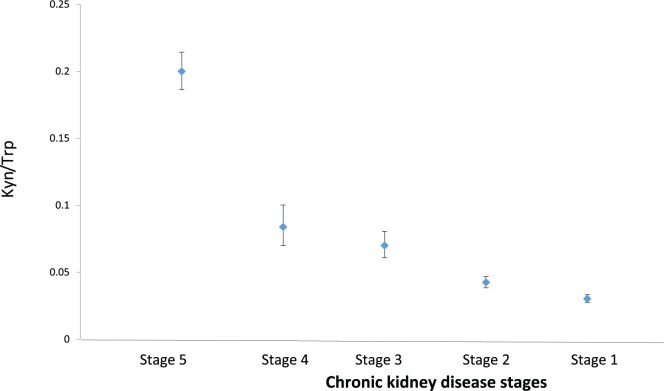
Kynurenine/tryptophan ratio by stages of chronic kidney disease. Data are geometric mean and 95% confidence interval. Statistical difference between stage 1 and stage 2: *P* = 0.03; stage 2 and stage 3: *P* = 0.003; stage 3 and stage 4: *P* = 0.43; stage 4 and stage 5: *P* < 0.0001.

The correlations between proinflammatory cytokines, eGFR, and TRP metabolites are shown in Table 3. The relationship between TRP and eGFR was remarkably robust and positive (*P* < 0.0001). In contrast, TRP metabolites KYN, KYNA, and QA showed strong negative correlations with eGFR (*P* < 0.0001 for all). Among the measured parameters, TNF-α had the strongest association with KYN metabolites (*P* < 0.0001), followed by IL-6, whereas CRP did not show any significant correlation. Figure 2 shows a visual representation of the relationships between TRP metabolites and the stages of CKD modeled by a smooth function. Table 3 also shows significant associations between KYN/TRP ratio with BMI (*r* = −.33, *P* < 0.05), TNF-α (*r* = .73, *P* < 0.0001) and IL-6 (*r* = .35, *P* < 0.01), and eGFR (r = −.93, *P* < 0.0001). Using ANCOVA, we further examined the link between KYN/TRP ratio and the stages of CKD. Figure 3 depicts that each time the CKD stage progressed (except from CKD stages 3 to 4), there was a statistically significant increase in the KYN/TRP ratio independent of age, sex, BMI, and CRP.

**Table 3.** Spearman correlation coefficients of age, body mass index, and renal function with specific inflammatory markers and tryptophan metabolites.

Variable	Age	BMI	eGFR	CRP	TNF-a	IL-6	TRP	KYN	KYNA	QA
BMI	−0.33^b^									
eGFR	−0.2	0.35^b^								
CRP	−0.17	0.26	−0.09							
TNF-α	0.08	−0.17	−0.72^d^	0.06						
IL-6	0.17	−0.13	−0.41^b^	0.33^a^	0.33^a^					
TRP	−0.18	0.15	0.82^d^	−0.10	−0.56^d^	−0.37^b^				
KYN	0.02	−0.33^a^	−0.83^d^	0.18	0.72^d^	0.33^a^	−0.57^d^			
KYNA	0.04	−0.02	−0.64^d^	0.23	0.42^c^	0.33^a^	0.32^a^	0.59^d^		
QA	0.10	−0.29^a^	−0.85^d^	0.24	0.65^d^	0.49^d^	−0.67^d^	0.84^d^	0.72^d^	
KYN/TRP ratio	0.14	−0.28^a^	−0.93^d^	0.17	0.73^d^	0.35^b^	−0.83^d^	0.89^d^	0.67^d^	0.85^d^

Abbreviations: BMI, body mass index; CRP, C-reactive protein; eGFR, estimated glomerular filtration rate; IL-6, interlerukin-6; KYN, kynurenine; KYNA, kynurenic acid; QA, quinolinic acid; TNF-α, tumor necrosis factor-α; TRP, tryptophan.

a *P* < 0.05; ^b^*P* < 0.01; ^c^*P* < 0.001; ^d^*P* < 0.0001.

Multiple linear regression analysis was used to examine the relation of inflammatory markers to KYN/TRP ratio and KYN taking into consideration the effect of important covariates such as age, sex, BMI, and eGFR (Table 4). Tumor necrosis factor-α and IL-6 were directly associated with KYN/TRP ratio after the adjustment for age, sex, and BMI (*P* < 0.001 even with the Bonferroni correction). Interleukin-6 was independently related to neither KYN/TRP ratio nor KYN after the additional adjustment for eGFR. Τumor necrosis factor-α was not related to KYN/TRP ratio after the additional adjustment for eGFR but was associated with KYN (*P* = 0.010). However, *P* value was attenuated to marginal significance (*P* = 0.060) after the Bonferroni correction. Age, sex, BMI, and eGFR explained 66.9% and 90.4% of variability of KYN concentration and KYN/TRP ratio, respectively. Adding TNF-α to the model accounted for an additional 3.9% of the KYN variance and 0.0% of the KYN/TRP ratio variance.

**Table 4.** Multiple linear regression models of the relationships between KYN/TRP and KYN (dependent variables) and inflammatory markers (independent variables).

	KYN/TRP			KYN		
	β-Coefficient	SE	*P* value	β-Coefficient	SE	*P* value
CRP (independent variable)						
Unadjusted	0.16	0.11	.163	0.11	0.07	.123
Adjusted for age, sex, and BMI	0.24	0.10	.023	0.17	0.07	.014
Adjusted for age, sex, BMI, and eGFR	−0.02	0.04	.700	0.04	0.05	.442
TNF-α (independent variable)						
Unadjusted	0.43	0.09	<.0001	0.30	0.05	<.0001
Adjusted for age, sex, and BMI	0.38	0.09	<.0001	0.27	0.05	<.0001
Adjusted for age, sex, BMI, and eGFR	0.02	0.04	.550	0.12	0.04	.010
IL-6 (independent variable)						
Unadjusted	0.36	0.09	.0004	0.18	0.06	.004
Adjusted for age, sex, and BMI	0.31	0.09	.002	0.15	0.06	.014
Adjusted for age, sex, BMI, and eGFR	−0.02	0.04	.572	−0.02	0.05	.740

Abbreviations: BMI, body mass index; CRP, C-reactive protein; eGFR, estimated glomerular filtration rate; IL-6, interleukin-6; KYN, kynurenine; SE, standard error; TNF-α, tumor necrosis factor-α; TRP, tryptophan.

Dependent and independent variables are log transformed.

The prevalence of albuminuria is highly variable in patients with CKD with T2D but is often considered as an important predictor of CKD progression—The higher the albuminuria, the greater the risk for kidney function.^25^ We analyzed data according to the degree of albuminuria using the standard definition^21^ and CKD stages 1 to 4; however, the number of subjects in each subgroup was extremely small, which precluded the generation of any meaningful result. Therefore, the analysis (age- and sex-adjusted) was limited only to degree of albuminuria, which revealed a significantly higher KYN/TRP ratio in subjects with macroalbuminuria (−2.75 ± 0.09, *P* < 0.001) and microalbuminuria (−2.95 ± 0.18, *P* < 0.05) than in patients with normoalbuminuria (−3.34 ± 0.08). The mean ± standard deviation eGFR in macroalbuminuria (n = 15), microalbuminuria (n = 4), and normoalbuminuria (n = 21) groups was 43.4 ± 6.1 (*P* < 0.001), 79.5 ± 11.7 (*P* < 0.01), and 96.4 ± 5.8 mL/min/1.73 m^2^, respectively.

## Discussion

Our study reports the first data on TRP metabolism in relation to inflammatory markers in patients with T2D and varying stages of CKD including ESKD. The results demonstrate a proportional and significant depletion of circulating TRP with the loss of kidney function. Compared with stage 1 CKD, plasma TRP levels were nearly 60% lower at stage 5 CKD. Tryptophan reduction was accompanied with an increase in the levels of KYN, KYNA, and QA—this preliminary observation may indicate the inductions of TRP degrading enzymes. Circulating levels of TRP metabolites positively and robustly correlated not only with the severity of kidney function but also with proinflammatory cytokines TNF-α and IL-6.

TRP metabolism in patients with moderate to severe CKD,^26^ including ESKD undergoing hemodialysis,^27,28^ has previously been reported. These studies included CKD patients of heterogeneous etiologies and excluded patients with early stages of CKD. The gradual TRP depletion that was proportional to the advancing CKD stages found in our study is in sharp contrast with Schefold et al. (see Figure 2a)^26^ who reported unaltered TRP levels in CKD stages 3 to 5—an unconventional finding. Our data, however, regarding significant correlations of the TRP metabolites and KYN/TRP ratio with severity of kidney function, are consistent with the results by the aforementioned study^26^ and a recent study by Zhang et al (see Figure 2).^29^ The overall findings, however, of the latter study^29^ that involved type 2 diabetic patients with impaired kidney function are difficult to interpret. Like Schefold et al.,^26^ the study did not find any difference in TRP levels between patients with eGFR greater than 60 and 31 to 60 mL/min/1.73 m^2^ (or CKD stage 3). It should be noted that eGFR greater than 60 mL/min/1.73 m^2^ includes both CKD stages 1 and 2. Importantly, Zhang et al^29^ did not measure any inflammatory marker or specify the number of subjects in each group of diabetic patients with eGFR of >60 and <60 ml/min/1.73 m^2^. Besides, the analytical method for TRP and KYN quantification and whether levels of TRP and KYN required log transformation remain unknown.

The underlying mechanism of accelerated TRP metabolism in CKD patients with T2D has not been investigated. Obviously, kidneys are the primary organs responsible for the elimination of TRP and its metabolites.^30^ TRP is effectively reabsorbed in the glomeruli, and reabsorption of KYN is significantly influenced by its plasma concentrations, ie, higher excretion fractions at increasing circulating levels.^30^ In CKD patients, this phenomenon is not observed,^31^ resulting in the accumulation of KYN in the circulation. Saito et al.^32^ demonstrated in nephrectomized animal model of CKD that in addition to diminished excretion, KYN accumulation could be due to enhanced synthesis and/or reduced metabolism.

We found enhanced IDO1 activity during the early stage of CKD (stage 2 vs stage 1, *P* < 0.01; Table 2). This novel observation suggests that upregulation of IDO1-mediated TRP metabolism may be an intrinsic feature of CKD. During chronic inflammation, IDO1 is upregulated and oxidatively degrades the indole ring of TRP to formylkynurenine, which subsequently yields KYN; once synthesized, KYN undergoes further metabolism through 3 distinct pathways to form several downstream intermediary metabolites (Figure 1)—a prevailing concept.^10,33^ Consistent with this notion, our data show striking positive correlation between TRP metabolites and proinflammatory cytokines (Table 3). It is interesting to note that animal studies^32,34,35^ consistently showed remarkable upregulation of TDO causing TRP depletion with concomitant KYN elevation. In addition, these studies^34,35^ noted unchanged tissue IDO1 activity in rats with experimental CKD compared with control group. One recent study^36^ identified TDO-induced TRP metabolism in patients with CKD and T2D. Therefore, TRP degradation via TDO activation in this patient population is conceivable.

The relationship between proinflammatory cytokines and IDO1 activity (KYN/TRP ratio) in CKD seems complex and is not well investigated. First, circulating proinflammatory cytokines show substantial variability, which apparently depends on the kidney function^37^ as impaired kidney function affects the clearance of cytokines. Second, it should also be emphasized, as mentioned previously, that handling of TRP and KYN by the kidneys is altered in CKD setting.^31,32^ Third, the reported associations between TRP and its metabolites with eGFR in the literature are inconsistent. For example, Goek et al.^38^ did not find any correlation between eGFR change with either TRP or KYN. In contrast, Solini et al.^36^ reported positive and inverse relationship of eGFR with TRP and KYN, respectively. Fourth, it is important to consider that TNF-α alone is not sufficient to induce IDO1 as shown by in vivo experiments, but it is an important potentiator of IDO1 expression in several different contexts.^7,39^ Finally, emerging data convincingly show that TDO also mediates immunoregulatory effects.^40^ Taken together, it is perhaps the combination of TDO and IDO1, not IDO1 alone, which is responsible for the accelerated metabolism of TRP in CKD. In light of the above discussion, the validity of KYN/TRP as an index of IDO1 activity needs reassessment in CKD and T2D context—a view that has recently been proposed by Badawy.^41^ It is relevant to acknowledge the potential involvement of indoleamine 2,3-dioxygenase 2 (IDO2) in TRP metabolism.^42^ IDO2 expression is predominant in the kidneys (as well as in antigen-presenting cells and a few other cell lines).^43,44^ While the biological role of IDO2 is yet to be fully elucidated, several studies point to IDO2 as an important contributor to TRP catabolism and production of KYN. For example, Ball et al.^43^ hypothesized that in conjunction with TDO expression in the liver, IDO2 may regulate plasma TRP by catabolizing excess TRP reabsorbed by the kidney tubules. In addition, like IDO1, IDO2 expression is upregulated in response to interferon-γ in several cell lines,^45^ which contradicts with previous study findings.^43^ The data reported in our study are unable to distinguish the relative contributions of these 3 enzymes on global changes in TRP catabolism along the KYN pathway.

We found accelerated IDO1 activity (KYN/TRP ratio) in patients with impaired kidney function with concomitant macroalbuminuria. Well-designed studies are warranted to replicate this novel finding and to elucidate the underlying biological mechanism because dietary TRP supplementation holds promise to ameliorate albuminuria as demonstrated by Kaysen and Kropp^46^ in animal model of CKD.

The stimulating preliminary data, nevertheless, presented here suggest that accelerated TRP metabolism via the KYN pathway may have significant contributions to CKD initiation, progression, and other symptomatology in patients with T2D. Of note, accelerated TRP metabolism is often implicated to fatigue, depression, and other psychiatric disorders,^47^ which are prevalent in advanced CKD.^48^ Moreover, our regression analysis suggests that primarily two distinct mechanisms (inflammation and impaired kidney function) contribute to the accumulation of KYN. This observation is potentially important because targeting inflammation alone may not be effective in reducing KYN levels and explains why hemodialysis alone is inefficient in removing KYN from the circulation of ESKD patients.^26^ Therefore, the KYN pathway may offer a viable new avenue for therapeutic intervention to address CKD-associated comorbidities in patients with T2D.

The present study is limited by the cross-sectional analysis of data from a relatively small sample size, particularly in stage 4 CD owing to extremely low prevalence and extraordinary mortality rate.^49,50^ We did not measure TDO or glucagon, and neurobehavioral phenotypes were not ascertained. In the parent study,^18^ plasma HbA1c was not available for all subjects. Recommended^20^ modest dietary animal protein restriction in patients with CKD with eGFR less than 60 mL/min/1.73 m^2^ may contribute to the lower level of TRP; however, it seems unlikely that dietary intake would affect circulating levels of TRP and or KYN/TRP ratio.^23^ The validity of the results in our study, however, is strengthened with the consistent findings of the positive relationship of TRP with eGFR and an inverse association between KYN and eGFR reported in a patient population similar to ours.^36^ Moreover, results of the current study are in agreement with the findings from a prospective study^51^ that showed significant association of KYN with eGFR progression.

In the aggregate, the study findings demonstrate the robust association between TRP metabolism with concomitant rise in several bioactive kynurenines in type 2 diabetic patients with impaired kidney function. The key metabolite KYN correlated significantly with circulating TNF-α independent of eGFR, although the strength of correlation attenuated to marginal significance (*P* = 0.060) after Bonferroni correction. These preliminary data provide a strong rationale for additional studies to determine the influence of TRP metabolites on the progression of diabetic kidney disease and overwhelming symptoms burden in this population. Future prospective studies should determine any temporal relationship of TRP metabolites with the progression of CKD stages and clarify the true cause of TRP depleting and kynurenines accumulation by measuring simultaneous direct assessment of TDO2 and both rate-limiting enzymes IDO1 and TDO.

## Supplementary Material

Supplementary material
