# Supplementary material for: Tryptophan Metabolism in Patients With Chronic Kidney Disease Secondary to Type 2 Diabetes: Relationship to Inflammatory Markers
Source: Int J Tryptophan Res. 2017 Mar 10;10:1178646917694600. doi: 10.1177/1178646917694600 (PMC5398653; doi:10.1177/1178646917694600)
Supplement: Supplementary material [file TRY694600_Supplementarydata_CLN.doc]

**Supplementary data**

**Supplemental Table**: Stages of chronic kidney disease in adults (Reference 20)

| **Stage** | **Description** | **eGFR (mL/min/1.73 m^2^)** |
| --- | --- | --- |
| **1** | Kidney damage with normal or ↑ eGFR | ≥90 |
| **2** | Kidney damage with mild ↓ eGFR | 60-89 |
| **3** | Moderate ↓ eGFR | 30-59 |
| **4** | Severe ↓ eGFR | 15-29 |
| **5** | Kidney failure | <15 (or dialysis) |

eGFR, estimated glomerular filtration rate
